# Reproduction of Large-Scale Bioreactor Conditions on Microfluidic Chips

**DOI:** 10.3390/microorganisms7040105

**Published:** 2019-04-19

**Authors:** Phuong Ho, Christoph Westerwalbesloh, Eugen Kaganovitch, Alexander Grünberger, Peter Neubauer, Dietrich Kohlheyer, Eric von Lieres

**Affiliations:** 1 Institute of Bio- and Geosciences, IBG-1: Biotechnology, Forschungszentrum Jülich, 52425 Jülich, Germany; p.ho@fz-juelich.de (P.H.); c.westerwalbesloh@fz-juelich.de (C.W.); e.kaganovitch@fz-juelich.de (E.K.); alexander.gruenberger@uni-bielefeld.de (A.G.); d.kohlheyer@fz-juelich.de (D.K.); 2 Multiscale Bioengineering, Bielefeld University, 33615 Bielefeld, Germany; 3 Bioprocess Engineering, Department of Biotechnology, Technische Universität Berlin, 13355 Berlin, Germany; peter.neubauer@tu-berlin.de; 4 Microscale Bioengineering, Aachener Verfahrenstechnik (AVT.MSB), RWTH Aachen University, 52074 Aachen, Germany

**Keywords:** microfluidics, single-cell analysis, modelling, simulation, computational fluid dynamics, frequency response, life line, monolayer growth chamber, mother machine, negative dielectrophoresis

## Abstract

Microbial cells in industrial large-scale bioreactors are exposed to fluctuating conditions, e.g., nutrient concentration, dissolved oxygen, temperature, and pH. These inhomogeneities can influence the cell physiology and metabolism, e.g., decelerate cell growth and product formation. Microfluidic systems offer new opportunities to study such effects in great detail by examining responses to varying environmental conditions at single-cell level. However, the possibility to reproduce large-scale bioreactor conditions in microscale cultivation systems has not yet been systematically investigated. Hence, we apply computational fluid dynamics (CFD) simulations to analyze and compare three commonly used microfluidic single-cell trapping and cultivation devices that are based on (i) mother machines (MM), (ii) monolayer growth chambers (MGC), and (iii) negative dielectrophoresis (nDEP). Several representative time-variant nutrient concentration profiles are applied at the chip entry. Responses to these input signals within the studied microfluidic devices are comparatively evaluated at the positions of the cultivated cells. The results are comprehensively presented in a Bode diagram that illustrates the degree of signal damping depending on the frequency of change in the inlet concentration. As a key finding, the MM can accurately reproduce signal changes that occur within 1 s or slower, which are typical for the environmental conditions observed by single cells in large-scale bioreactors, while faster changes are levelled out. In contrast, the nDEP and MGC are found to level out signal changes occurring within 10 s or faster, which can be critical for the proposed application.

## 1. Introduction

Industrial bioprocesses, for example the production of the antibiotic penicillin by the fungal species *Penicillium chrysogenum*, typically involve suspension cultivation of the microorganisms in stirred large-scale bioreactors with working volumes up to several hundred cubic meters [1]. Within those large-scale bioreactors, spatial gradients and highly dynamic inhomogeneous conditions are caused by imperfect mixing due to limited power input and consequently, the cultivated microorganisms are exposed to fluctuating pH, temperature, nutrients and dissolved oxygen [2,3]. Studies have shown that even short-time exposure to dynamic environmental changes can influence cell physiology and thus overall process performance [2,4].

Such bioprocesses are typically designed by optimizing strains and process conditions in laboratory-scale bioreactors to benefit from lower cost, higher throughput, and parallelization. However, processes transferred to industrial scale often show unpredictable behavior such as performance loss regarding biomass formation and product substrate yield. This behavior is likely caused by the unavoidable inhomogeneities in large-scale bioreactors which are not present at the development scale [5]. Several studies have employed lab-scale cultivation systems with multiple compartments to simulate and study the impact of such large-scale inhomogeneities [3,4,6,7]. For instance, Enfors et al. [2] have observed cellular responses on mRNA level within only 14 s after a glucose pulse. Microbes have further been reported to metabolically react to glucose pulses after only 2 s by forming significantly high amounts of by-products such as acetate and formate under aerobic and anaerobic conditions [4].

### 1.1. Large-Scale Bioreactor Conditions

It has thus become of great interest to understand large-scale bioreactor inhomogeneities and to couple them with cell population models to establish a link between bioreactor conditions and cell physiology [8,9]. Haringa et al. [10] have applied coupled hydrodynamic Euler-Lagrange CFD simulations to determine the trajectories of individual *P. chrysogenum* cells, also referred to as lifelines, passing through different regimes of substrate availability (starvation, limitation and excess) in a 54 m${}^{3}$ bioreactor. Moreover, Haringa et al. [9] have reported that the microbial cells can travel from regions with substrate excess to regions with substrate deficiency and vice versa within 1 s to 10 s. Hence, environmental fluctuations typically occur in the same time frame as the resulting cellular responses.

### 1.2. Microfluidic Single-Cell Analysis

**Figure 1:**
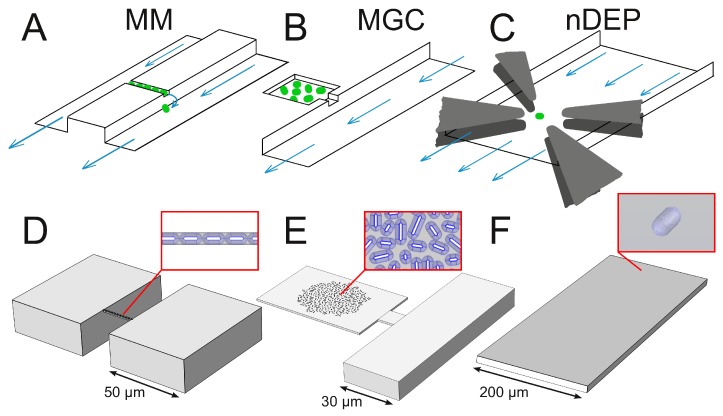
Investigated microfluidic trapping and cultivation devices: (**A**) mother machine (MM) with growth channel connected to two supply channels, (**B**) monolayer growth chamber (MGC) connected to one supply channel, and (**C**) negative dielectrophoresis (nDEP) within supply channel. Cells are indicated by green dots and fluid flow by blue arrows. (**D**–**F**) illustrate respective CFD geometries. (**F**) features an extended channel after the cell trap to reduce the impact of computational artefacts at the exit boundary. Red insets show microcolonies in geometries (**D**,**E**), geometry (**F**) contains a single cell.

Microfluidic single-cell analysis is a new method to investigate individual cellular behavior [11,12,13]. Former studies have found striking cell-to-cell heterogeneity with respect to specific growth rate [14], cell morphology [15], gene expression via fluorescence reporter proteins [16] and cell viability [17]. Microfluidic single-cell cultivation allows precise control of environmental conditions, as comparatively few cells are cultivated and fast laminar medium flow (see also Appendix A) in large supply channels enables effective provision of fresh nutrients and removal of products [13]. This facilitates the investigation of causal relations between environmental conditions and phenotypes, also heterogeneity in connection with fluorescent reporters [16], which can be used for studying single-cell responses to fluctuating environment typically encountered in large-scale bioreactors [18]. Several microfluidic cell-trapping and cultivation concepts can potentially be used for this purpose, in particular based on mother machines (MM), monolayer growth chambers (MGC), and negative dielectrophoresis (nDEP) (see Figure 1). To date most microfluidic cultivations apply constant nutrient supply. Moreover, oscillating nutrient concentrations and defined changes in the range of minutes to hours has been reported in recent years [19,20].

#### 1.2.1. Mother Machine

Wang et al. [21] have developed the MM to study ageing effects and to analyze long-term growth and division of *Escherichia coli*. The original MM design consists of one supply channel, which is continuously perfused with fresh medium, and from which multiple dead-ended growth channels are branching off. Long et al. [22] have designed a modified version in which the growth channels are situated between two symmetric supply channels (see Figure 1A). Each growth channel can harbor multiple cells (around ten) and restricts growth to one direction. Cells emerging from the growth channel are removed by the flowing fluid within the supply channels. MM have since their invention found numerous applications, for example to investigate growth dynamics (growth rate, cell size and gene expression) at single-cell level and population responses to dynamic feed perturbations in the hour range [17,20,22].

#### 1.2.2. Monolayer Growth Chamber

The MGC design allows the study of small cell populations in a monolayer. The cells are mechanically trapped in a growth chamber and are only able to grow in two spatial dimensions (see Figure 1B). MGCs are applied for examining growth dynamics, e.g., growth rate [23], division characteristics [24], morphology [24] and gene expression [25] for colonies of up to several hundred cells. Gradients of nutrients and excreted metabolites can occur and result in variations of cell sizes and cellular behavior, in particular for large and densely populated chambers [26].

#### 1.2.3. Negative Dielectrophoresis

Using the nDEP technique, individual cells can be trapped in the center of the supply channel within continuous medium flow. It is based on a non-uniform electric field, achieved by applying alternating current to an octupole arrangement of electrodes (see Figure 1C) [27]. nDEP allows isolation of individual cells and to cultivate them without any mechanical contact. However, the flow rate in the trapping region of the device is limited by the force required to resist the flow drag. The applied voltage pulses cause joule heating, excess levels of which can become harmful to microbial cells and negatively affect their viability and physiology [28].

### 1.3. Scope

In this contribution we study if the dynamics of environmental conditions typically encountered by individual cells in large-scale bioreactors can be reproduced in the trapping zones of microfluidic chips. The above introduced devices, MM, MGC and nDEP, are systematically compared by computational approaches with respect to their capability for exposing cultivated cells to nutrient concentration profiles. Specifically, a step change, a representative life line determined by Haringa et al. [9], and oscillations with a wide range of frequencies are used.

## 2. Materials and Methods

### 2.1. Computational

CFD was used to predict the stationary velocity field as well as the transient mass transfer within the studied devices [26,29]. Calculations were performed using COMSOL Multiphysics 5.3a (COMSOL AB, Stockholm, Sweden). The maximum solute concentration was 222 mmol L${}^{-1}$ of glucose, equivalent to the defined CGXII growth medium [30]. At these low concentrations, the flow field is practically independent of the concentration field, and consequently both these fields can be computed sequentially. The boundary probe feature of COMSOL Multiphysics was used for determining average substrate concentrations at the surfaces of observed cells. All computational geometries were meshed with unstructured tetrahedral elements. The standard (normal) element size in COMSOL (see also Appendix C) was found to be sufficient after performing mesh independence studies for all three geometries.

#### 2.1.1. Fluid Dynamics

The Navier-Stokes equations [31] for an incompressible, isothermal and Newtonian fluid were solved for the stationary velocity field:

(1) $$\begin{array}{cc} \mu \nabla^{2}u & =\nabla p \end{array}$$

(2) $$\begin{array}{cc} \nabla \;\cdot \;u & =0 \end{array}$$

Here, u denotes fluid velocity vector in $m\;s^{-1}$, ρ fluid density in $kg\;m^{-3}$, *p* fluid pressure in Pa, and μ dynamic viscosity in Pas. The liquid in the channels and chambers was modelled with the density and viscosity of pure water, i.e., ρ = 995.6 kg $m^{-3}$ and μ = $7.97\times 10^{-4}$ Pa s as reported by Comesaña et al. [32]. The no-slip condition was applied at PDMS and glass walls. The parabolic velocity profile at the inlets of the supply channels was established using the laminar inflow feature of COMSOL with an inflow length of 100 μm. The average inflow velocity $v_{in}$ was $6.66\times 10^{3}$ μm $s^{-1}$ for MM, $1.88\times 10^{3}$ μm $s^{-1}$ for MGC, and 10 μm $s^{-1}$ for the nDEP system. Zero pressure was specified as outlet boundary condition of the supply channels.

#### 2.1.2. Mass Transport

The mass transport was modelled using the diffusion-advection equation according to Deen [31]:

(3) $$\frac{\partial c}{\partial t}+\nabla \;\cdot \;\left(-D\nabla c\right)+u\;\cdot \;\nabla c=0$$

Here, *c* denotes solute concentration in mmol $L^{-1}$, *D* the binary diffusion coefficient of the solute in water in m^2^ $s^{-1}$, and u the velocity vector in m $s^{-1}$. The diffusion coefficient of glucose is $5.4\times 10^{-10}$ $m^{2}$ $s^{-1}$ [33].

The time dependent inlet concentrations of the supply channels are defined in Section 2.1.3, and vanishing diffusive flow was used at their outlets. Nutrient consumption by the cells is modelled using a flux boundary condition at the cell surfaces. Dependence of the uptake rate, *Q* in mmol m^−2^ s^−1^, on glucose concentration, *c* in mmol L^−1^, is described by Monod’s equation [34]:

(4) $$Q=\hat{Q}\frac{c}{c+K_{S}}$$

Wendisch et al. [35] have reported a $K_{S}$-value of 4.5 mmol L^−1^ for *C. glutamicum* and glucose. The glucose uptake rate per cell dry weight $q_{G}$ = 4.42 mmol g^−1^ h^−1^ and the single-cell dry weight of $1.5\times 10^{-12}$ g reported by Unthan et al. [30] for *C. glutamicum* were used to calculate an uptake rate $\hat{Q}$ of $1.82\times 10^{-7}$ mol $m^{-2}s^{-1}$.

#### 2.1.3. Inlet Signals

Three different input signals were used. The first signal is a step change from 0 to 222 mmol L^−1^ at t= 1 s. To facilitate numerical integration, the discontinuous step was approximated by a smoothed linear transition within 0.1 s. The second signal is adopted from large-scale bioreactor simulations by Haringa et al. [9] and features typical variations in nutrient availability. The published data is densely sampled and then interpolated by a piecewise cubic polynomial. For comparison with the first signal, the dimensionless life line is scaled to a maximum concentration of 222 mmol L^−1^. The third signal is a sine wave with the amplitude 222 mmol L^−1^ and a variable frequency *f*:

(5) $$c_{in}\left(t\right)=222\mathrm{mmol}L^{-1}\left(\frac{1}{2}\;\cdot \;\sin\left(2\pi \;\cdot \;f\;\cdot \;t-\frac{\pi }{2}\right)+\frac{1}{2}\right)$$

#### 2.1.4. Signal Analysis

For the third signal, a frequency response analysis was performed by computing the amplitude ratio A${}_{out}$/A${}_{in}$ between the chip inlet and the cell surfaces for stationary oscillations. The system was considered stationary when the amplitudes varied less than 1% over four consecutive oscillations. The analysis was performed for frequencies between 100 Hz and 0.001 Hz, which equals periods between 0.01 s and 1000 s, and visualized in a Bode plot.

#### 2.1.5. Computational Geometry

The MM was modelled following Long et al. [22] (see Figure 1A,D). The growth channel is 1 μm wide, 20 μm long and 1 μm high. Both sides of the growth channel are connected to adjacent supply channels which are 50 μm wide, 70 μm long (begins 50 μm upstream of the growth channel) and 20 μm high. The growth channel is completely filled with 11 cells.

The MGC was adapted from Probst et al. [36] (see Figure 1B,E). An asymmetric design with only one supply channel was chosen. The chamber is 40 μm wide, 60 μm long and 1 μm high. It is connected to a supply channel through an entrance that is 10 μm wide, 9 μm long and 1 μm high. The adjacent supply channel is 30 μm wide, 100 μm long (begins 50 μm upstream of the chamber entrance) and 10 μm high. The chamber is filled with a typical colony of 300 cells. In two alternative designs, (1) the width of the entrance is extended to 40 μm, and (2) the chamber is connected to a second supply channel.

The nDEP was modelled following Dusny and Schmid [37] (see Figure 1C,F). The trapping region is 200 μm wide, 50 μm long, and 12 μm high. After the trapping zone, the channel is extended by 500 μm in order to diminish effects caused by the outlet boundary condition, as back diffusion cannot be neglected at the typical nDEP flow rates below 10 μm s^−1^ [27]. A single cell is placed in the center of the channel.

In all three devices, cellular parameters specific for the bacterium *C. glutamicum* were used for modelling the cells. The cells are described as cylinders with spherical ends and a radius of 0.52 μm with an overall length of 2 μm. The cells are modelled as inaccessible volumes with sink terms at their surfaces, as described in Section 2.1.2. For the colonies, the cell in the center was used for probing the glucose concentration. The inlet boundary condition of the supply channel was defined in a distance of 50 μm of the growth channel (MM), chamber entrance (MGC), or cell (nDEP).

### 2.2. Experimental

**Figure 2:**
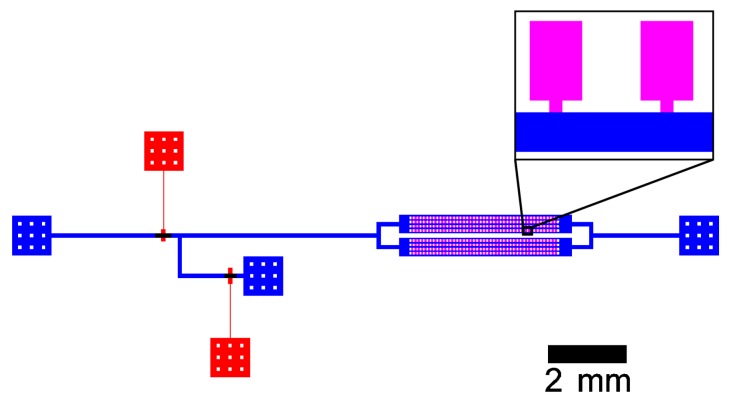
Schematic channel layout of the microfluidic chip with supply channels (blue), growth chambers (pink), and control channels (red). The magnification shows two adjacent MGC.

The microfluidic validation experiment was conducted in a polydimethylsiloxane (PDMS)-based device. Figure 2 shows the schematic design of the chip. The supply channel is connected to two inlet streams that are controlled by pneumatic valves as described by Unger et al. [38]. It has a width of 100 μm and the same height as the computational MGC model, but the distance between the junction of the inlet streams and the entrance of the growth chamber is approximately 9 mm. The dimensions of growth chambers and entrances are identical to the computational MGC model.

#### 2.2.1. Device Fabrication

The fabrication process follows a previously described multilayer soft lithography protocol [38,39], with parameters listed in the Appendix D. Two master layers were used, one for the channel structures (bottom layer) and another for the pneumatic valves (top layer). The top layer was made by casting a 4 mm thick layer of PDMS, using base and curing agent in a ratio of 7:1. The bottom layer was created by spinning a thin layer of PDMS on the bottom mold at 2300 rpm, using base and curing agent in a ratio of 20:1. After separate curing at 80 ${}^{\circ }C$, the top layer was released from the mold and aligned to the bottom layer. Bonding of the two layers was achieved by curing at 80 ${}^{\circ }C$ for 60 min. After releasing the bonded PDMS device from the wafer, inlet and outlet holes were punched. The device was bonded to a glass substrate by oxygen plasma treatment. More details on chip fabrication can be found in the Appendix E.

#### 2.2.2. Device Operation and Microscopic Imaging

The microfluidic experiment was observed using an automated inverted microscope (Ti-E, Nikon, Tokyo, Japan) with 20×/0.75 objective (MRD70200, Nikon, Tokyo, Japan), YFP fluorescence filter (ex:  500 nm, em:  542 nm, F36-528, AHF Analysentechnik, Tübingen, Germany) and high resolution camera (Andor Luca-R DL-880, Oxford Instruments, Belfast, UK). The chip was illuminated using a sola light engine (Lumencor, Beaverton, OR, USA) at 9% intensity. Images were taken with frequency 12.4 Hz and exposure time 80 ms. The inlet streams of the observed supply channel were connected to water and fluorescein solution reservoirs, respectively, and the outlet to a waste vessel with tubing (Tygon AAD04103, Saint-Gobain, Paris, France) and connectors (Precision tips, Nordson, Erkrath, Germany). Pressured air was used to operate the valves and to drive water or fluorescein solution (1 mmol L^−1^, F6377, Sigma-Aldrich, St. Louis, MO, USA) through the channel. A pressure of 2 was used for closing the valves and 50 mbar to drive the flows, both controlled by a pressure controller (MFCS-EZ, Fluigent, Lowell, MA, USA). The experiment was performed by switching from water to fluorescein solution after ca. 90 s and back to water approximately 80 s later. It was stopped after 240 s. The inlet signal was created by applying air pressure to one or the other control channel while keeping both inlet streams under constant pressure.

#### 2.2.3. Data Analysis

**Figure 3:**
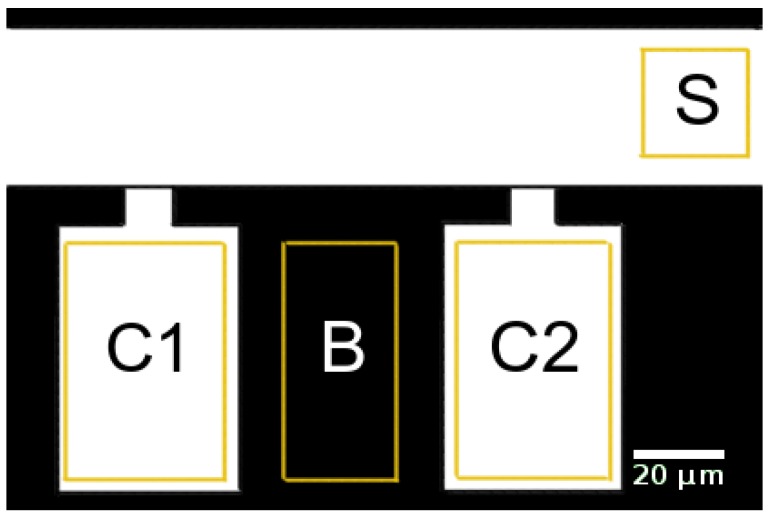
Regions of interest for data analysis in supply channel (S), growth chambers (C1, C2), and background region (B).

The average brightness within the supply channel, the cultivation chambers, and a control region between these chambers was determined by image analysis using the “region of interest” functionality of Fiji (ImageJ version 1.52e as described by Schindelin et al. [40]). The analyzed regions are indicated by yellow rectangles in Figure 3. The spatially averaged signals were corrected for background brightness by subtracting the overall time average of all signals until 0.5 s before the first switch. The spatially averaged signals in the chambers were further corrected for background brightness by subtracting the spatially averaged signal in the control region between these chambers (region B in Figure 3). Then, all signals were normalized by scaling their maxima to 1. The input signal for the simulation was created from the corrected supply channel signal (region S in Figure 3). The signal until 0.5 s before the first switch was set to zero, and the other times were interpolated using splines (scipy.interpolate.UnivariateSpline in python) yielding the inlet signal for the simulation.

## 3. Results and Discussion

The devices are compared with respect to their ability to reproduce large-scale bioreactor conditions using different input signals.

### 3.1. Step Change

**Figure 4:**
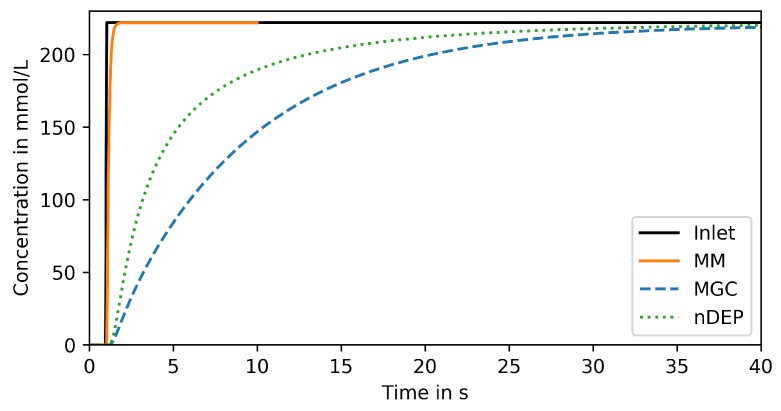
Reproduced signal in trapping region of compared devices for step change input.

First, the response to a step concentration change at the system inlet is analyzed. Figure 4 shows the response, spatially averaged over the surface of a representative cell in the center of the trapping region of the respective device, over time. Naturally, a fast response is desired but only its steepness is relevant as a shift in time can easily be accounted for in the single-cell analysis procedure.

Only the MM can reproduce the signal within 1 s (orange solid line in Figure 4), which is the response time required for reproducing large-scale bioreactor conditions (see Section 1.1). More specifically, 95% of the input amplitude are reached within 0.3 s. Both other devices reproduce the signal much less steeply. The nDEP (green dotted line in Figure 4) reaches 95% of the input amplitude after 18 s and the MGC (blue dashed line) even later, after 26 s.

These results indicate that only the MM is suitable for reproducing large-scale bioreactor conditions on microfluidic chips. This might seem counterintuitive as in the nDEP the cell is placed in the center of the main supply channel. However, this device must be operated at much lower flow rates (typically below 10 μm s^−1^ [27]), at which convection is no longer dominant in the main channel, as shown in the Appendix B. Consequently, steep signals are flattened out, similar to band broadening effects in chromatography devices. Larger flow rates would require increasing the nDEP force above a limit where the captured cells are stressed and potentially harmed by the joule heating induced by the applied alternating electrical fields [28].

In the MGC, mass transfer is limited by the diffusive transport from the supply channel into the chamber. This can be improved by enlarging the entrance and/or connecting the chamber to a second supply channel, as will be further studied in Section 3.4.

### 3.2. Life Line

**Figure 5:**
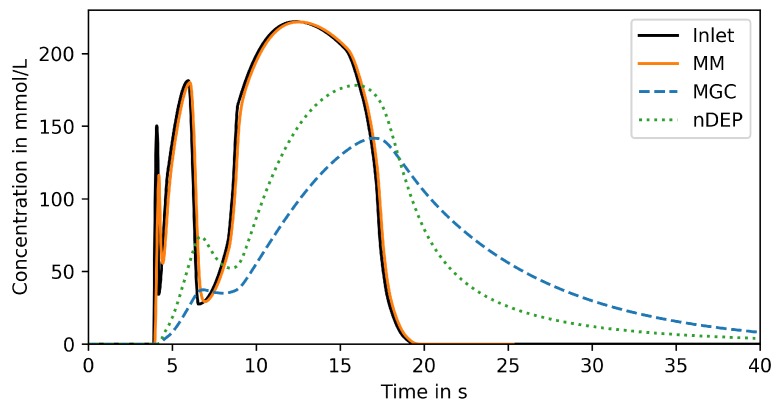
Reproduced signal in trapping region of compared devices for life line input.

The results of the previous section are confirmed by analyzing the response to a typical life line observed in a large-scale bioreactor, which has been simulated by Haringa et al. [9]. As shown in Figure 5, the signal features three concentration peaks with increasing width and amplitude. The MM can tightly reproduce the entire signal. Both nDEP and MGC completely miss the first peak and can by far not reproduce the amplitude of the second peak (44% for nDEP and 17% for MGC). The nDEP captures the third peak better than the MGC, but both are clearly not satisfying even for a peak width of about 10 s.

### 3.3. Frequency Response Analysis

**Figure 6:**
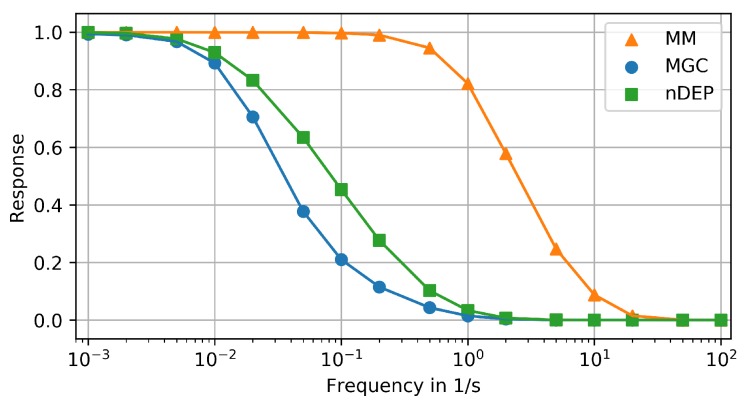
Frequency amplitude response diagram of compared devices: (1) MM (orange triangles), (2) MGC (blue dots), (3) nDEP (green squares).

The results of the previous section indicate that the studied devices differ in their capacity to reproduce signals with increasing period (peak width). This is now systematically analyzed using sine signals with a range of frequencies as input. Similar to a Bode diagram from control theory, Figure 6 shows the ratio of signal amplitudes between the chip inlet and the cells in the center of the respective trapping region over the logarithm of the frequency (reciprocal period) [41]. As discussed before, time/phase shifts are not investigated in this study.

According to Figure 6, the MM can reproduce signals with a period of down to 5 s (frequency of up to 0.2 Hz) with an amplitude loss of less than 5%. The cutoff frequency, at which the amplitude starts to drop significantly, is 0.2 Hz. Signals with a period of down to 0.1 s (frequency of up to 10 Hz) are reproduced with an amplitude loss of up to 95%. This information could potentially be used for specifically amplifying signal components between 1 Hz and 10 Hz after performing a Fourier transformation. However, reproducibility of signals with periods of 1 s and above suffices for studying the impact of large-scale bioreactor conditions on microfluidic chips (Section 1.1).

The nDEP and the MGC (Figure 6) dampen the amplitude of signals with periods below ca. 200 s (frequencies above ca. 0.005 Hz) by more than 5%. The cutoff frequencies of both these devices is 0.005 Hz. Both systems reproduce signals with a period of down to ca. 1 s (frequency of up to ca. 1 Hz) with an amplitude loss of up to 99%. Hence, an amplification of specific frequencies might help to use these devices for single-cell analysis under relevant dynamic conditions. The MGC has also been simulated with only one cell, located in the center of the chamber. For all input signals (step, life line, frequency response), the results with a single cell or colony did not differ significantly. Hence, the results for a colony are shown and discussed here. They represent typical operating conditions of the device.

### 3.4. Design Optimization

As mentioned in Section 3.1, the MGC design can potentially be improved by enlarging the entrance and by connecting the chamber to a second supply channel. The first measure increases the cross-sectional area of the entrance region through which solute molecules are exchanged between the supply channel and the growth chamber. The second measure has the same effect and additionally shortens the path length of diffusive transport within the chamber. In addition, fabrication tolerances of the microfluidic chip can cause slight asymmetries in chamber geometry and consequently some convective flow through the chamber. Convective transport can be beneficial but is hardly controllable and might even cause cells to be flushed out of the chamber. Hence this effect is excluded from the current study.

**Figure 7:**
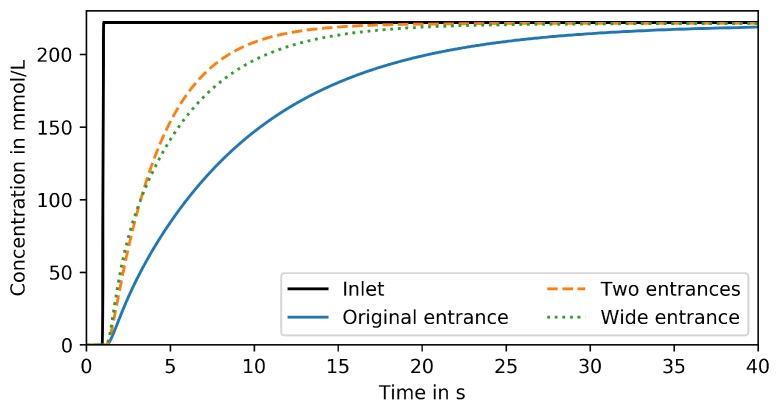
Reproduced signal in trapping region of MGC devices with varied entrance size and position for step change input.

Figure 7 compares responses to the step signal from Section 3.1 in a MGC with (i) original entrance with 10 μm width, (ii) enlarged entrance width 40 μm width and (iii) two entrances with 10 μm width. Both measures, enlarging the single entrance and adding a second entrance, have similar effects. 95% of the inlet concentration are reached 11 s after the step change which is almost 56% faster than the original design. However, even the improved MGC is by far not capable of reproducing dynamic signals in the frequency range required for studying large-scale bioreactor conditions.

### 3.5. Experimental Validation

**Figure 8:**
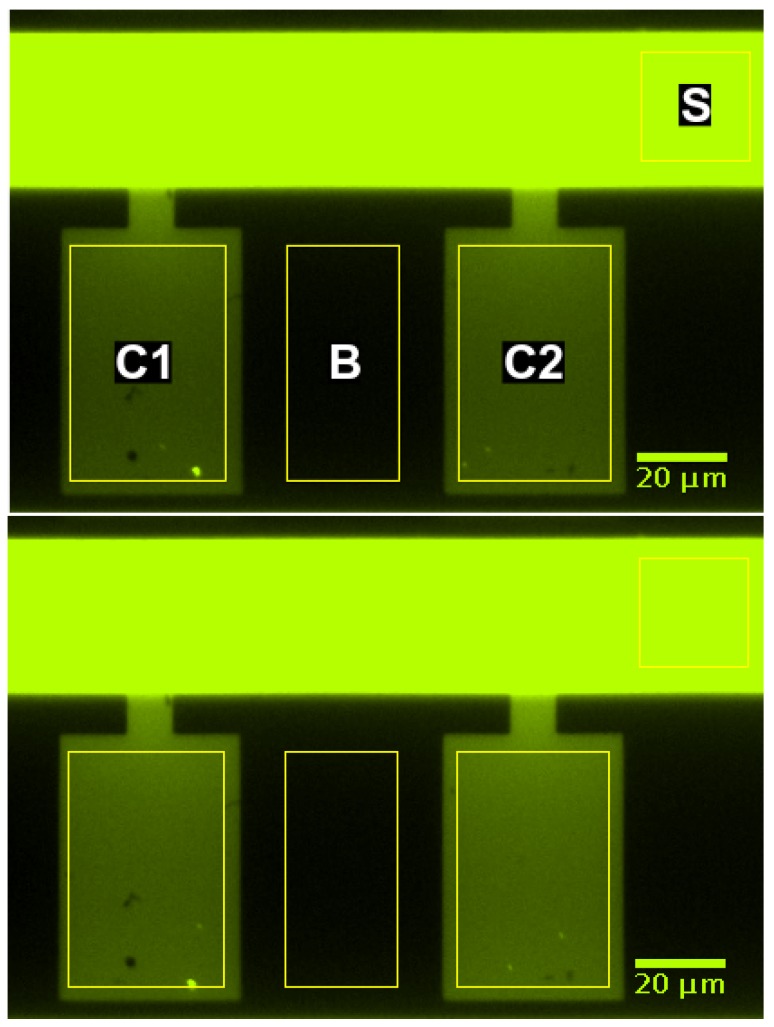
Fluorescence signal in supply channel (S), growth chambers (C1, C2), and background region (B) at t=42s (**top**) and t=80s (**bottom**).

**Figure 9:**
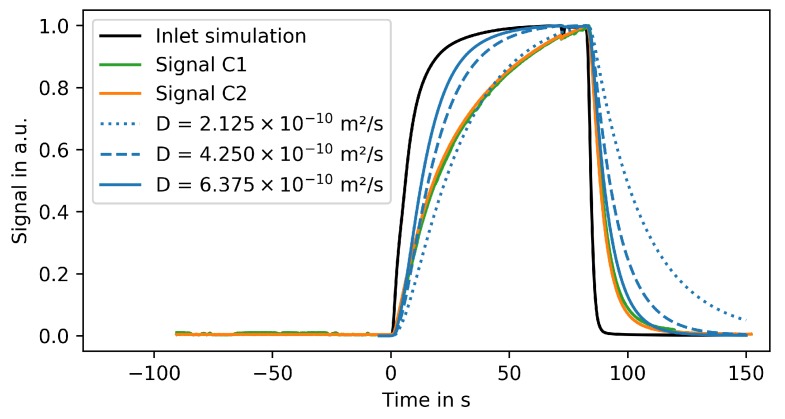
Spatial average of measured fluorescence signals in different regions of the microfluidic chip and simulated concentration profiles for the same chamber geometry using different diffusion rates.

The MGC was chosen for experimentally validating the computational approach. Figure 8 shows the fluorescence signal of two growth chambers and a section of the supply channel at one point in time. The fluorescence signal is spatially averaged over the marked regions and corrected for background brightness as described in Section 2.2.3. The supply channel is fed from the right-hand side. Due to the high liquid velocity in the supply channel, both growth chambers are exposed to practically the same entrance signal. Consequently, both chambers show almost the same intensity profile over time (see Figure 9). The time t=0s marks the switch from water to fluorescein.

The spline interpolated signal in region S of Figure 8 is used as inlet boundary condition for the corresponding CFD simulation (black line in Figure 9). As the experimental data, the simulation results are spatially averaged over the growth chamber and normalized such that the maximal signal is 1. The system was simulated using a published diffusion coefficient of $4.25\times 10^{-10}$ $m^{2}$ $s^{-1}$ [42] for fluorescein. For comparison, simulations were also performed using 0.5 and 1.5 times this diffusion coefficient.

Figure 9 shows that the experimental results can be properly reproduced by these simulations. However, the increasing part of the signal is more accurately described using the lower diffusion coefficient while the decreasing signal is better reproduced using the larger coefficient. These discrepancies can be caused by several factors: First, the correlation between the fluorescence signal and the fluorescein concentration is not perfectly linear. The signal can also be influenced by the height of the respective region on microfluidic chip. Finally, the applied background correction does not account for light scattering, surface effects, and quenching.

## 4. Conclusions and Outlook

The aim of this work was to investigate three different single-cell microfluidic devices for their characteristics in reproducing pulse-based environmental changes as they are thought to occur in large-scale industrial bioreactors. This would provide the opportunity to directly observe metabolic and gene expression responses to such environmental fluctuations on a single-cell level, which is not possible within bioreactors. Extreme scale-down experiments on microfluidic chips with cell-trapping devices can be a viable alternative, provided that dynamic signals can be reproduced on the relevant time scale in the order of one second.

Here this important issue was studied for three frequently used devices that are based on (i) MM, (ii) MGC, and (iii) negative dielectrophoresis (nDEP). CFD simulations were performed using a step function, a representative life line and sine waves with a range of frequencies as input signals. The latter are comprehensively analyzed using a frequency amplitude response diagram. This so-called Bode diagram [41] is not commonly used in biotechnology but as we have demonstrated is particularly useful for comparing microfluidic systems. The computational analysis allows the avoidance of tedious and futile experiments and to focus the experimental effort on feasible and promising setups.

Counterintuitively, the nDEP cannot reproduce input signals in the required frequency range, even though the cells are trapped in the center of the supply channel. This can be explained by the fact that the flow velocity is strongly limited by the applicable nDEP force and, consequently, the device cannot be operated in the convection dominant regime. In contrast, the supply channels of the MM and MGC are operated in the convection dominant regime. However, both devices require additional diffusive transport from the supply channel to the trapped cells. Only in the MM the path length of this diffusive transport is short enough to allow for reproduction of signals with down to 1 Hz, as required for studying the impact on these fluctuations on cellular behavior, whereas the other studied devices have nearly a complete (up to 99%) signal loss for 1 Hz. Although MGC can be improved by enlarging the entrances and using a second supply channel, the optimized device still dampens relevant frequencies.

In conclusion, only the MM allows the quantitative study of the impact of typical reactor inhomogeneities on single cells which provides important information for developing strains with robust performance under production conditions.

## Appendix A. Laminar Flow

The Reynolds number describes the ratio of inertial and viscous forces in a flowing liquid. In the supply channels of the studied microfluidic chips it is defined by:

(A1) $$Re=\frac{\rho \;\cdot \;v\;\cdot \;D_{h}}{\mu }$$

In Equation (A1), ρ denotes the fluid density in $kgm^{-3}$, *v* the fluid velocity in m s^−1^, $D_{h}$ the diameter of the supply channel in m, and μ the dynamic viscosity in kg m^−1^ s^−1^. Reynolds numbers below 1 are typical for microfluidic systems and indicate laminar flow. For the studied devices, Re${}_{MM}$ = 0.24, Re${}_{MGC}$ = 0.04 and Re${}_{nDEP}$ = 2.8 × 10^−7^.

## Appendix B. Dominant Mass Transfer

The Péclet number describes the ratio of convection and diffusion in a flowing liquid. It is defined by:

(A2) $$Pe=\frac{Lu}{D}$$

In Equation (A2), *L* denotes the characteristic transport distance in m, *u* the flow velocity in m s^−1^, and *D* the diffusion coefficient in m^2^ s^−1^.

Péclet numbers well below 1 indicate diffusion dominant mass transport and well above 1 indicate convection dominant mass transfer. Péclet numbers around 1 indicate that both mechanisms significantly contribute to mass transfer. In the supply channels of the studied devices, the Péclet number is 617 for the MM, 174 for the MGC, and 0.93 for the nDEP. In the growth channels/chambers of the MM and MGC, the Péclet numbers are 0.02 and 0.07, respectively.

## Appendix C. Mesh and Uptake Model Details

The standard mesh (normal) had 60,134 elements and an average element quality of 0.6451 for the MM geometry, 54,713 elements and an average element quality of 0.6144 for the MGC geometry, and 210,736 elements and an average element quality of 0.6976 for the nDEP geometry.

Single-cell uptake rates obtained by literature review differ significantly from each other and are obtained from bulk measurements. Therefore, for a worst-case study, test simulations were performed with ten-fold increased values. The results differed by less than 1%, which is similar to the remaining error determined in the mesh independence study.

## Appendix D. Process Parameters Photolithography

### Appendix D.1. Top Layer Master Mold Fabrication

1. Wafer dehydration in an oven at 200 ${}^{\circ }C$ for 10 min
2. Spin-Coating of SU-8 photoresist (SU-8 2010, Microchemicals GmbH, Ulm, Germany) at 3500 rpm. Final resist thickness: 10 μm
3. Soft bake on a hotplate at 65 ${}^{\circ }C$ for 3.5 min, then at 95 ${}^{\circ }C$ for 3.5 min
4. UV light exposure (Karl Suss MA4, Süss Micro Tec, Garching bei München, Germany) for 4 s (wavelength, soft vacuum contact mode)
5. Post exposure bake on a hotplate at 65 ${}^{\circ }C$ for 3 min, then at 95 ${}^{\circ }C$ for 3 min
6. Two-Step development in mr-Dev 600 (Microchemicals GmbH, Ulm, Germany) for 45 s + 60 s, then cleaning in 2-Propanol for 20 s
7. Hardbake on a hotplate at 150 ${}^{\circ }C$ for 6 h

### Appendix D.2. Bottom Layer Master Mold Fabrication

1. Wafer dehydration in an oven at 200 ${}^{\circ }C$ for 10 min
2. Spin-Coating of SU-8 photoresist (SU-8 2000.5 and 2010, mixing ratio 12:88) at 2000 rpm. Final resist thickness: 1 μm
3. Soft bake on a hotplate at 65 ${}^{\circ }C$ for 1 min, then at 95 ${}^{\circ }C$ for 1 min
4. UV light exposure (Karl Suss MA4, Süss Micro Tec, Garching bei München, Germany) for 2.8 s (wavelength, vacuum contact mode)
5. Post exposure bake on a hotplate at 65 ${}^{\circ }C$ for 1 min, then at 95 ${}^{\circ }C$ for 1 min
6. Two-Step development in mr-Dev 600 (Microchemicals GmbH, Ulm, Germany) for 30+30s, then cleaning in 2-Propanol for 20 s
7. Hardbake on a hotplate at 150 ${}^{\circ }C$ for 10 min
8. Spin-Coating of SU-8 photoresist (SU-8 2010) at 3500 rpm. Final resist thickness: 10 μm
9. Soft bake on a hotplate at 65 ${}^{\circ }C$ for 3.5 min, then at 95 ${}^{\circ }C$ for 3.5 min
10. UV light exposure (Karl Suss MA4, Süss Micro Tec, Garching bei München, Germany) for 4 s (wavelength, soft vacuum contact mode)
11. Post exposure bake on a hotplate at 65 ${}^{\circ }C$ for 3 min, then at 95 ${}^{\circ }C$ for 3 min
12. Two-Step development in mr-Dev 600 for 45s+60s, then cleaning in 2-Propanol for 20 s
13. Hardbake on a hotplate at 150 ${}^{\circ }C$ for 6 h
14. Application of hexamethyldisilazane (HDMS) adhesion promoter
15. Spin-Coating of SPR 220-7 photoresist (Microchemicals GmbH, Ulm, Germany) at 3000 rpm. Final resist thickness: 7 μm
16. Soft bake on a hotplate at 105 ${}^{\circ }C$ for 4 min
17. UV light exposure (Karl Suss MA4, Süss Micro Tec, Garching bei München, Germany) for 1 min (wavelength, soft vacuum contact mode)
18. Post exposure bake on a hotplate at 65 ${}^{\circ }C$ for 1 min, then at 95 ${}^{\circ }C$ for 1 min
19. Two-Step development in MF-D 26 (Dow Chemical, Midland, MI, USA) for 1 min
20. Hardbake on a hotplate at 120 ${}^{\circ }C$ for 15 min

## Appendix E. Soft Lithography and Multilayer Chip Assembly

The fabrication process for a similar chip has been previously described in detail [39].

1. Preparation of a 7:1 (base:curing agent) mixture of PDMS (Sylgard 184 Silicone Elastomer kit, Dow Corning Corporation, Midland, MI, USA)
2. Preparation of a 20:1 (base:curing agent) mixture of PDMS
3. Degassing of both mixtures in a desiccator for 30 min
4. Top layer molding
  (a) Application of the 7:1 PDMS mixture on the top layer master mold
  (b) Baking in an oven at 80 ${}^{\circ }C$ for 40 min
  (c) Releasing hardened PDMS mold
  (d) Cutting chips, punching inlets
5. Bottom layer molding
  (a) Application of the 20:1 PDMS mixture on the bottom layer master mold
  (b) Spin-coating at 2300 rpm for 60 s, ramp 200 rpm/s
  (c) Relaxation time 20 min
  (d) Baking in an oven at 80 ${}^{\circ }C$ for 20 min
6. Layer bonding
  (a) Alignment of the top layer to the bottom layer under a microscope
  (b) Bonding in an oven at 80 ${}^{\circ }C$ for 1 h
7. Chip bonding
  (a) Release of the bonded PDMS layers from the bottom layer master mold
  (b) Punching inlets and outlets using a punching tool (size: 0.75 mm, World Precision Instruments, Sarasota, FL, USA)
  (c) Bonding of the PDMS chips to a glass substrate (170 μm) by exposure to oxygen plasma (Femto Plasma Cleaner, Diener Electronics, Ebhausen, Germany) at an oxygen flow rate of 20 sccm for 25 s
